# From Nurse‐Led to Clinical Dietitian‐Led Nutritional Care in Hospital Practice: Changes in Nutritional Risk Screening Rates, Patient Nutritional Intake and Professional Perspectives

**DOI:** 10.1111/jhn.70363

**Published:** 2026-10-08

**Authors:** Marianne Boll Kristensen, Ingeborg Krarup Rask, Lene Nissen Møller Schmidt, Kirstine Guld Frederiksen, Alexander Erichsen, Lone Viggers

**Affiliations:** ^1^ Department of Nutrition Gødstrup Hospital Herning Denmark; ^2^ NIDO, Centre for Research and Education Gødstrup Hospital Herning Denmark; ^3^ Department of Medicine Gødstrup Hospital Herning Denmark

**Keywords:** clinical dietitian, interdisciplinary collaboration, nurse, nutritional care, nutritional risk screening

## Abstract

**Introduction:**

Daily nutritional care is often nurse‐led, with clinical dietitians involved only upon referral. This study explored the effects of integrating clinical dietitians into daily nutritional care through reallocation of nutritional care tasks at a medical pulmonary ward, focusing on nutritional risk screening practices, patient nutritional intake, and professional perspectives.

**Methods:**

This multimethod study included three substudies: (1) a retrospective cohort analysis of nutritional risk screening practices, (2) a quasi‐experimental comparison of nutritional intake between a historical control group and the intervention group, and (3) qualitative interviews with nurses and clinical dietitians following completion of the intervention.

**Results:**

In substudy 1, 1678 admissions were analysed. During clinical dietitian‐led nutritional care, 55% of patients were screened within 24 h of admission and 76% overall, compared to 29% and 43% during nurse‐led care. In substudy 2, a greater proportion of patients in the intervention group achieved ≥ 75% of their energy requirements than in the control group (79% vs. 57%, *p* = 0.023), whereas differences in protein intake were not statistically significant (50% vs. 35%). In substudy 3, both professions reported improved nutritional care and collaboration. Nurses described relief but reduced awareness of patients' nutrition, while clinical dietitians found the new tasks meaningful but challenging. Coordination and dialogue were important in adapting to changed routines.

**Conclusion:**

Reallocating nutritional care tasks from nurses to clinical dietitians was associated with improved nutritional risk screening, higher energy coverage, and stronger interdisciplinary collaboration, particularly when supported by close coordination and dialogue between clinical dietitians and nursing staff.

## Summary

- Assigning nutritional risk screening and nutritional care planning to clinical dietitians may improve the timely identification and management of patients at nutritional risk.
- Daily ward presence of clinical dietitians can strengthen interdisciplinary collaboration, support continuity of nutritional care, and enable closer patient monitoring.
- Close coordination, collaboration and ongoing dialogue between clinical dietitians and nursing staff are essential when reorganising nutritional care.

## 1 Introduction

Malnutrition among hospitalised patients is associated with prolonged hospital stay, poorer prognoses, reduced quality of life, and increased mortality [1, 2, 3]. The organisation of nutritional care is therefore an important determinant of patient outcomes. Effective nutritional care includes early identification and management of patients at nutritional risk, and routine nutritional risk screening has long been recommended in international guidelines [4, 5]. Despite this, and despite strong evidence that nutritional interventions improve outcomes [6], a gap persists between guidelines and clinical practice, with studies repeatedly reporting incomplete screening practices, insufficient follow‐up, and/or inadequate nutritional intake among patients at nutritional risk [2, 7, 8].

In many healthcare systems, including Denmark's, daily nutritional care is usually nurse‐led. This includes tasks such as nutritional risk screening, estimation of nutritional requirements, initiation of appropriate nutritional interventions, and assistance with meal ordering. However, nurses juggle numerous responsibilities, and studies show that nutrition is often deprioritised for more urgent tasks [9, 10, 11, 12]. Although clinical dietitians have specialised expertise in nutritional assessment and intervention, they are typically involved only upon referral. As a result, if nutritional risk screening is not performed or the need for nutritional intervention is not recognised, clinical dietitians may not be involved, limiting patients' access to specialised nutritional support.

This traditional task allocation may reflect both the constant presence of nurses on wards and the limited integration of clinical dietitians into routine care. A recent Danish quality assurance study found that closer integration of clinical dietitians into routine ward care was associated with improvements in nutritional screening and patient outcomes [13]. However, evidence remains limited regarding the effects of such organisational changes and how they are experienced by the healthcare professionals involved. This study aimed to explore the potential effects of integrating clinical dietitians into daily nutritional care through the reallocation of nutritional care tasks from nurses to clinical dietitians. Objectives were to: (1) assess adherence to nutritional risk screening guidelines during periods of clinical dietitian‐led and nurse‐led nutritional care, (2) compare patient nutritional intake between the two models, and (3) explore nurses' and clinical dietitians' experiences of the reallocation and their perspectives on optimal task allocation.

## 2 Materials and Methods

### 2.1 Study Design and Setting

The study was conducted at a 27‐bed medical pulmonary ward in a Danish hospital, where daily nutritional care tasks were reallocated from nurses to clinical dietitians.

This multimethod study combined quantitative and qualitative methods across three substudies. Substudy 1 evaluated nutritional risk screening guideline adherence during and after the intervention through a retrospective cohort analysis. Substudy 2 assessed the effect on patient nutritional intake using a quasi‐experimental design. Substudy 3 explored how nurses and clinical dietitians experienced the reallocation through qualitative interviews. Reporting followed relevant guidelines for observational, non‐randomised, and qualitative studies [14, 15, 16].

The intervention, including reallocation of nutritional care tasks and daily ward presence of a clinical dietitian, lasted from March 2022 to July 2023, with data collected before, during and after (Figure 1).

**Figure 1:**
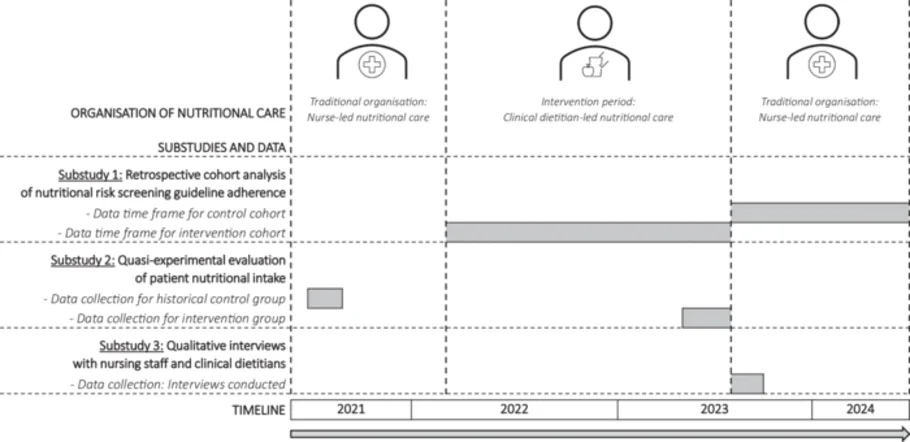
Study overview.

### 2.2 Usual Care: Nurse‐Led Nutritional Care

In the traditional task allocation, daily nutritional care is the nurses' responsibility. Local guidelines require that patients with an expected stay of ≥ 48 h are screened within 24 h of admission, using the Nutritional Risk Screening 2002 (NRS 2002) [17]. Screening is documented using a dedicated electronic screening form in the electronic patient record. Patients identified as being at nutritional risk (≥ 3 points) must have a nutritional care plan with estimated requirements, prescribed diet, and defined goals. The foodservice is á la carte with electronic meal ordering. Each patient has an electronic tablet allowing them to view the menu for their prescribed diet and place meal orders. The electronic system also functions as a nutritional intake record, displaying all ordered food items. The proportion consumed of each item can be recorded using plate diagrams (0%, 25%, 50%, 75% or 100%), while additional foods and beverages consumed outside the electronic ordering system can also be entered. Based on these registrations, the system automatically calculates daily energy and protein intake. Meals can be ordered between 7.30 am and 7.30 pm. Outside these hours, nursing staff can retrieve snacks and lighter meals from the ward kitchen for patients. Patients unable to order food themselves are assisted by nursing staff, who also provide mealtime assistance when needed. The á la carte concept and the electronic system are described in detail elsewhere [18].

Clinical dietitians are not systematically present on the ward and are involved only upon referral of patients by physicians or nurses (under delegated authority). Following referral, the clinical dietitian attends the ward within 1–3 days, most often within 24 h, and provides individualised nutritional treatment, structured according to the Nutrition Care Process [19]. If considered relevant, follow‐up is conducted after 1–3 days during hospitalisation and/or via telephone after discharge.

### 2.3 The Intervention: Clinical Dietitian‐Led Nutritional Care

During the intervention period, responsibility for nutritional risk screening, nutritional care planning and initiation of nutritional care was transferred to clinical dietitians, while nurses retained responsibility for overall patient care and mealtime assistance (practical assistance to consume the served meal). A clinical dietitian was present on the ward each day (including weekends and holidays) from 7.00 am to 2.30 pm. During this time, the clinical dietitian was responsible for conducting nutritional risk screening, developing nutritional care plans, participating in nursing conferences, and assisting patients with meal ordering regardless of their nutritional risk. The clinical dietitian was also responsible for nutritional risk screening of patients admitted after 2.30 pm on the following day. In the event of illness, another clinical dietitian from the nutrition department replaced the assigned dietitian as soon as possible, while nursing staff assisted with breakfast meal ordering until the replacement arrived.

The clinical dietitian still provided individualised nutritional treatment; however, rather than relying on referrals, the clinical dietitian independently performed nutritional risk screening, identified patients requiring nutritional intervention, and thereby led decisions regarding nutritional care.

The intervention was primarily delivered by five clinical dietitians. Each typically worked on the ward for 2–3 consecutive days, followed by days with other duties in the nutrition department. Additional clinical dietitians provided cover in the event of illness.

Nine beds were short‐term (expected stay of < 48 h), and clinical dietitians were not responsible for nutritional care (including nutritional risk screening) of patients in these beds, although referral remained possible.

### 2.4 Substudy 1: Nutritional Risk Screening Guideline Adherence

Data for the retrospective cohort analysis in substudy 1 were extracted from the electronic patient record system and included all admissions between 1 March 2022 and 30 June 2024, covering the intervention period and an 11‐month post‐intervention follow‐up. A post‐intervention follow‐up period was chosen rather than a pre‐intervention comparison period because a previous study [18], conducted on the ward immediately before the intervention, may have increased awareness of nutrition among ward staff and influenced usual practice. Data extraction was limited to June 2024 because the local nutritional risk screening guideline and the associated electronic screening form were revised from July 2024, introducing changes to the nutritional risk screening procedure that could have affected comparability over time. The extracted data did not distinguish between standard beds and short‐term beds within the ward. Consequently, admissions to short‐term beds could not be identified and excluded based on bed type. However, as the local guideline recommends nutritional risk screening only for patients with an expected length of stay ≥ 48 h, admissions with an actual length of stay < 48 h were excluded, meaning that most admissions to short‐term beds were likely removed from the dataset. Admissions with a length of stay ≥ 48 h were therefore considered eligible for inclusion. Patients registered as terminally ill at admission or within 48 h were excluded, as the local guideline allows nutritional risk screening to be omitted in terminally ill patients if nutritional intervention is not considered relevant. Readmissions within 7 days were also excluded, as the local guideline recommends repeat nutritional risk screening only after 7 days for patients not at nutritional risk. Including these admissions could therefore have resulted in guideline‐adherent care being classified as non‐adherence.

Data were extracted in pseudonymised form by a hospital data consultant. The dataset included a unique identifier allowing identification of multiple admissions for the same individual, while the research team had no access to information enabling identification of individual patients.

For each admission, it was assessed whether nutritional risk screening was conducted and whether it occurred within 24 h of admission. Details on screening criteria are provided in Supporting Information: File 1.

To assess differences between periods, a run chart of monthly screening proportions was constructed. Three standard rules for interpreting run charts were applied to detect non‐random variation over time: (1) a shift, defined as six or more consecutive points all above or all below the median; (2) a trend, defined as five or more consecutive points consistently increasing or decreasing; and (3) too few or too many runs, assessed by comparing the observed number to tabled critical values; a run is a series of points on one side of the median. These rules correspond to *p* < 0.05 [20].

### 2.5 Substudy 2: Nutritional Intake

Substudy 2 compared nutritional intake during the intervention with a historical control group from the same pulmonary ward, derived from a previous three‐arm quasi‐experimental study evaluating the hospital's à la carte foodservice concept [18]. In the previous study, intervention group 1 received à la carte foodservice combined with the usual organisation of nutritional care, in which nursing staff were responsible for nutritional risk screening and nutritional care and clinical dietitians were involved by referral. This group was compared with both a control group receiving the traditional hospital foodservice concept and a second intervention group receiving à la carte foodservice combined with individualised nutritional treatment by a clinical dietitian. Following the previous study, the à la carte foodservice concept was implemented throughout the hospital and was therefore part of usual care at the time of the present study. Intervention group 1 was selected as the historical control because it most closely reflected the combination of foodservice and organisation of nutritional care in place before the present intervention. A concurrent control group was not feasible because the organisational intervention was implemented across the entire ward. Although the hospital relocated to a newly built regional hospital following the merger of two hospitals in 2022, the ward's clinical function, patient population and core clinical staff remained largely unchanged. Blinding was not feasible due to the study design. Eligible patients were newly admitted, at nutritional risk (NRS 2002 ≥ 3), with an expected stay ≥ 48 h, and able to understand and speak Danish. The historical control group had corresponding eligibility criteria, except that an anticipated length of stay of ≥ 3 days was required. Exclusion criteria were conditions precluding informed consent (e.g. severe dementia), terminal illness, and need for special diets or enteral/parenteral nutrition. Patients with terminal illness were excluded because nutritional goals in this group are primarily palliative and may differ from those of the study population. Patients requiring special diets or enteral/parenteral nutrition were excluded to ensure comparability with the historical control group, as the previous study evaluated the à la carte foodservice concept only among patients receiving one of the two general diets for patients at nutritional risk (hospital diet or energy‐dense diet). Before approaching a patient, the research team member consulted the responsible nurse or clinical dietitian to ensure that the patient was considered able to provide informed consent. All eligible patients admitted between 15 May and 29 July 2023 were consecutively invited. No a priori sample size calculation was performed; all eligible patients admitted within a predefined recruitment period comparable to the control group were invited. Using the same procedures as in the previous study [18], nutritional intake during the first three full days of admission was recorded using the integrated electronic meal‐ordering and nutritional intake registration system. Daily meal orders and recorded intake were reviewed together with the participant by a research team member to ensure completeness. Based on the participant's report, the amounts consumed of ordered foods that had not yet been registered, as well as any foods or beverages consumed outside the electronic ordering system, were recorded. The electronic meal‐ordering and intake‐registration interface is illustrated in Supporting Information: File 2.

Patient‐level mean energy and protein intakes over the 3‐day period were calculated and expressed as percentages of their respective estimated requirements. For participants discharged early, available full days were included.

Energy requirements were estimated for each participant using the Harris‐Benedict equation for basal metabolic rate [21] multiplied by an activity factor (1.1 for bedridden participants, 1.2 for patients able to walk to the toilet but otherwise mostly bedridden, and 1.3 for participants able to walk around the ward) and a stress factor (1.0–1.3) according to disease severity. Protein requirements were estimated as 1.2–1.5 g/kg body weight/day, based on clinical judgement of disease severity (0.8–1.1 g/kg/day in renal impairment without renal replacement therapy). For participants with BMI > 27 kg/m^2^, requirements were calculated using the weight corresponding to BMI = 27 kg/m^2^. In the historical control group, nurses estimated requirements using standard table values (Supporting Information: File 3) [18]. Stata/MP 18.5 was used for analyses. Baseline characteristics were compared between the intervention and historical control groups using the Mann–Whitney *U* test for continuous variables and the Chi‐squared test or Fisher's Exact test for categorical variables, as appropriate.

Logistic regression models adjusted for sex, age, BMI, and NRS 2002 score estimated group‐wise proportions of participants achieving ≥ 75% of estimated energy and protein requirements, using the post‐estimation margins command. Between‐group differences were assessed with a post‐estimation Wald test. A significance level of 0.05 was applied. As a sensitivity analysis, energy requirements were recalculated for both groups using a standardised weight‐based method based on the Danish national guidance on nutritional risk and malnutrition management [22]: 113 kJ (27 kcal)/kg/day for participants with BMI < 30 kg/m^2^ and 85 kJ (20 kcal)/kg/day for participants with BMI ≥ 30 kg/m^2^. The adjusted analysis of the proportion achieving at least 75% of estimated energy requirements was then repeated using these recalculated requirements. As the guidance notes that energy and protein requirements may vary with low or high BMI and recommends an individual nutrition plan for patients with BMI < 18.5 kg/m^2^ [22], the analysis was repeated after excluding these participants. As this resulted in a larger between‐group difference, a post hoc analysis of the primary energy outcome was performed with the same exclusion to examine whether this pattern was also present using the original clinically estimated requirements. Protein requirements were similarly recalculated using fixed values of 1.2 and 1.3 g/kg/day to explore the impact of applying a common estimation method to both groups.

### 2.6 Substudy 3: Nurses' and Clinical Dietitians' Perspectives

Substudy 3 explored nurses and clinical dietitians' experiences through individual semi‐structured interviews. All five clinical dietitians who had been an integrated part of the intervention were invited. Nurses were eligible if they had worked at the ward both before and during the intervention. Approximately 30 nurses met this criterion during the interview period. Interview days were scheduled with the head nurse, who assisted in identifying nurses who met the predefined eligibility criterion and were available on those days. The number of interviews with nurses was determined pragmatically and informed by an ongoing assessment of data saturation, with recruitment ending when additional interviews were considered unlikely to yield substantially new insights. Participation was voluntary, and all individuals invited to participate were informed that declining participation would have no consequences for their employment. Written informed consent was obtained with only the participant and interviewer present, and willingness to participate was verbally reconfirmed before the audio recording was started.

Interviews were conducted during August and September 2023 by the first author, a female clinical dietitian with a PhD degree and qualitative research experience. Participants were informed that the purpose was to explore their experiences of the task reallocation. They were encouraged to speak openly about both positive and negative experiences and to raise issues they considered important, even if these were not covered by the interview guide. The clinical dietitians knew the interviewer as a colleague, whereas the nurses were generally not familiar with her; to them, she was introduced as the nutrition department's research and education coordinator. The interviewer had no managerial responsibility for any of the participants.

A semi‐structured interview guide was developed (Supporting Information: File 4), containing questions on experiences of collaboration and task reallocation; perspectives on task allocation in relation to expertise and competencies; and any additional reflections. Questions were largely the same for nurses and clinical dietitians, with minor adjustments in wording. The interview guide was approved by the head nurse and the head of nutrition. The individual semi‐structured interviews were conducted during work hours in an undisturbed room with only the interviewer and participant present. One clinical dietitian was interviewed via video call due to leave. Interviews were conducted in Danish, audio‐recorded and transcribed verbatim in Danish.

Data were analysed separately for nurses and clinical dietitians using qualitative content analysis [23] to derive central themes. First, two researchers (the first and second authors) independently listened to all audio recordings to gain an overall impression of the data. Next, they independently read through all transcripts, identifying relevant meaning units, which were then condensed and abstracted into the researchers' own words. Finally, the two researchers collaborated to categorise the coded units into subthemes and overarching themes. All coding and qualitative analysis were conducted using the Danish transcripts. No qualitative analysis software was used. The analysis process is illustrated in Supporting Information: File 5. Theme names and illustrative quotations were translated into English only for reporting. Translations were undertaken by the first author and reviewed by the second author, with any uncertainties resolved through discussion to preserve the conceptual meaning of the original data.

Both researchers conducting the analysis worked in the nutrition department – one as research and education coordinator, and the other as quality coordinator. Although the first author is a clinical dietitian, neither researcher provided nutritional care on the medical pulmonary ward during the intervention. All co‐authors approved the analysis results. Interview transcripts were not returned to participants for correction. Preliminary findings were presented at a staff meeting attended by the clinical dietitians. Participants were encouraged to raise any comments or additional perspectives during the discussion or, if preferred, afterwards directly with the first author. It was not feasible to arrange a corresponding meeting with the nurses, despite this being offered, and the findings were therefore presented to the management of the pulmonary medical ward. Neither the discussions nor any subsequent comments resulted in changes to the themes or interpretation of the data.

### 2.7 Ethical Considerations

Written informed consent was obtained from all participants in substudies 2 and 3. Participation was voluntary, and participants were informed that declining participation or withdrawing from the study would have no consequences for their treatment or employment. For substudy 1, pseudonymised routinely collected data were extracted from the electronic patient record system following approval by the hospital management. According to Danish legislation, this type of study does not require approval from a regional research ethics committee or informed consent from individual patients. Substudy 2 was registered with the Danish Data Protection Agency (j. no. 1‐16‐02‐174‐23), and based on a summary of the study protocol, the regional research ethics committee determined that the study did not require approval from the committee (j. no. 1‐10‐72‐6‐23). According to Danish legislation, interview studies do not require approval from a research ethics committee.

## 3 Results

### 3.1 Substudy 1: Nutritional Risk Screening Guideline Adherence

The dataset for substudy 1 contained data from 4094 admissions (2908 patients). Of these, 167 were excluded because of terminal illness, 293 were readmissions within 7 days, and 1956 lasted ≤ 48 h. This left 1,678 admissions involving 1406 patients for analysis (intervention period: 1020 admissions/874 patients; post‐intervention period: 658/598).

In the run chart (Figure 2), shifts (≥ 6 consecutive points above or below the median) and too few runs (< 10 for 27 data points outside the median) indicated that nutritional risk screening practices changed significantly from clinical dietitian‐led to nurse‐led nutritional care. During clinical dietitian‐led nutritional care, the median proportion of patients screened within 24 h was 55%, and 76% were screened overall. After the transition to nurse‐led nutritional care, these medians dropped to 29% and 43%. A sensitivity analysis including admissions with a length of stay < 48 h resulted in lower absolute screening proportions but showed the same temporal pattern and did not alter the overall interpretation of the findings (Supporting Information: File 6).

**Figure 2:**
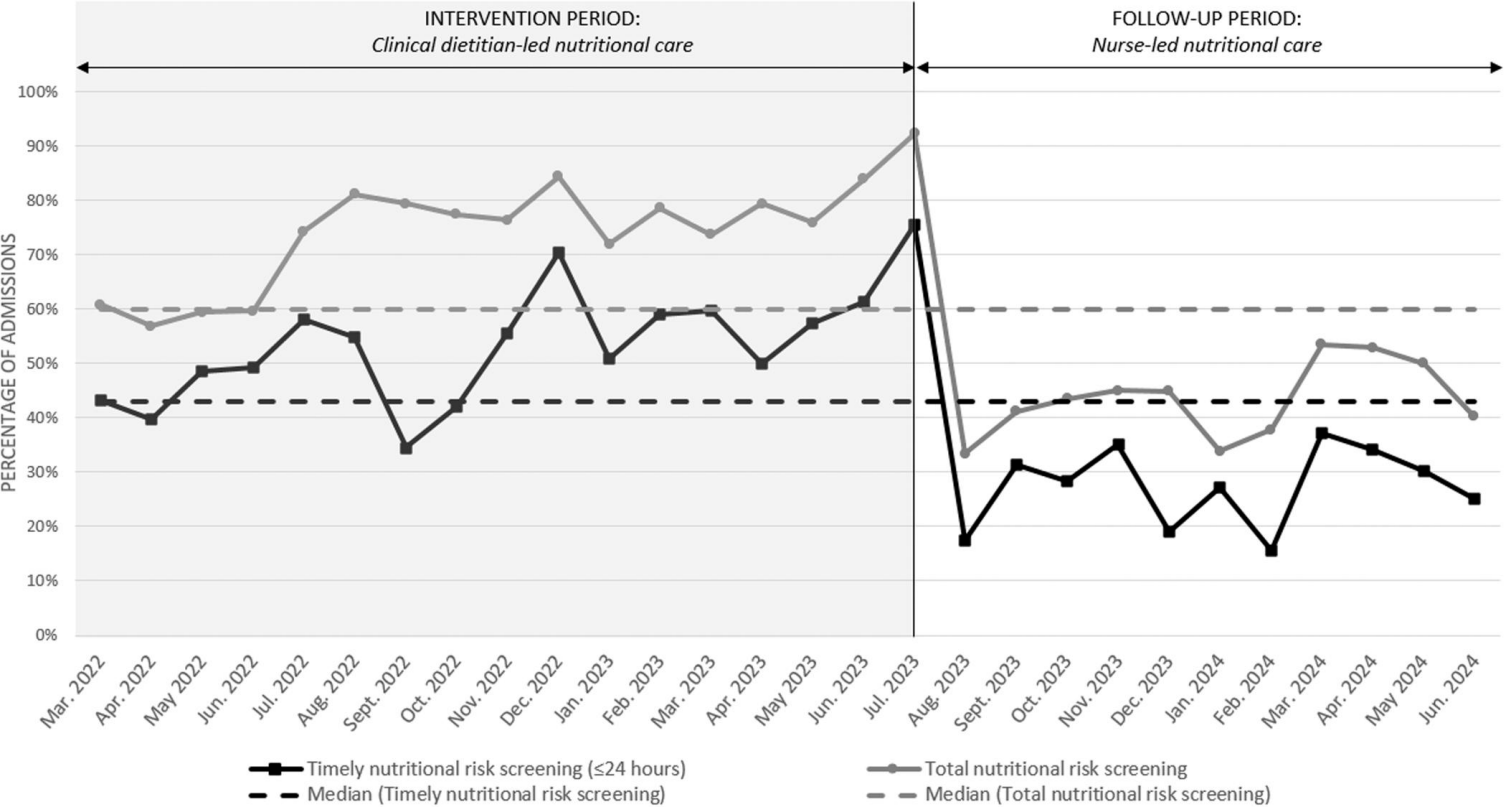
Run chart illustrating monthly adherence to nutritional risk screening guidelines. The run chart shows the monthly proportion of admissions with nutritional risk screening completed within 24 h of admission and overall. Admissions with a length of stay < 48 h were excluded. The vertical line indicates the end of the clinical dietitian‐led intervention period.

### 3.2 Substudy 2: Nutritional Intake

For substudy 2, 55 patients met the inclusion criteria and were invited to participate; 41 provided informed consent. Nine were subsequently excluded: three were declared terminal, five had been included despite transfer from another ward and therefore were not newly admitted, and one patient was transferred to another ward before completing a full day registration. Hence, 32 patients were included in analyses, compared with 68 in the historical control group. Table 1 shows participant characteristics. Length of stay was shorter in the intervention group, whereas no statistically significant differences were observed in baseline characteristics.

**Table 1:** Participant characteristics in substudy 2.

	Historical control group *n* = 68	Intervention group *n* = 32	Difference between groups *p*‐value^a^
Gender, *n* (%)			0.591
Male	28 (41%)	15 (47%)	
Female	40 (59%)	17 (53%)	
Age (years)			0.427
Median (Q1–Q3)	77 (71.5–84)	77.5 (66.5–83.5)	
Body Mass Index (kg/m^2^)			0.076
Median (Q1–Q3)	20.6 (18.5–24.4)	23.0 (19.3–27.1)	
NRS 2002 Score, *n* (%)			0.161
3 points	34 (50%)	17 (53%)	
4 points	16 (24%)	12 (38%)	
5 points	12 (18%)	3 (9%)	
≥ 6 points	6 (9%)	0	
Admission diagnosis, *n* (%)			0.265
COPD	29 (43%)	8 (25%)	
Pulmonary infections	22 (32%)	12 (38%)	
Lung cancer	2 (3%)	3 (9%)	
Dyspnoea	4 (6%)	1 (3%)	
Other diagnoses	11 (16%)	8 (25%)	
Length of hospital stay (days)			0.009
Median (Q1–Q3)	6 (4–10)	4 (3–6)^b^	

Abbreviations: COPD, chronic obstructive pulmonary disease; NRS 2002, Nutritional Risk Screening 2002.

^a^ Differences between groups were tested with Chi^2^ for gender, Fisher's Exact test for other categorical variables, and Mann‐Whitney *U* test for continuous variables.

^b^ *n* = 30, as two participants were transferred to other departments before hospital discharge.

The proportion of participants achieving a nutritional intake of ≥ 75% of their energy requirement was higher in the intervention group than in the control group (79% vs. 57%, *p* = 0.023). For protein intake, 50% of the intervention group versus 35% in the control group reached ≥ 75% of requirements, although this difference was not statistically significant (Figure 3).

**Figure 3:**
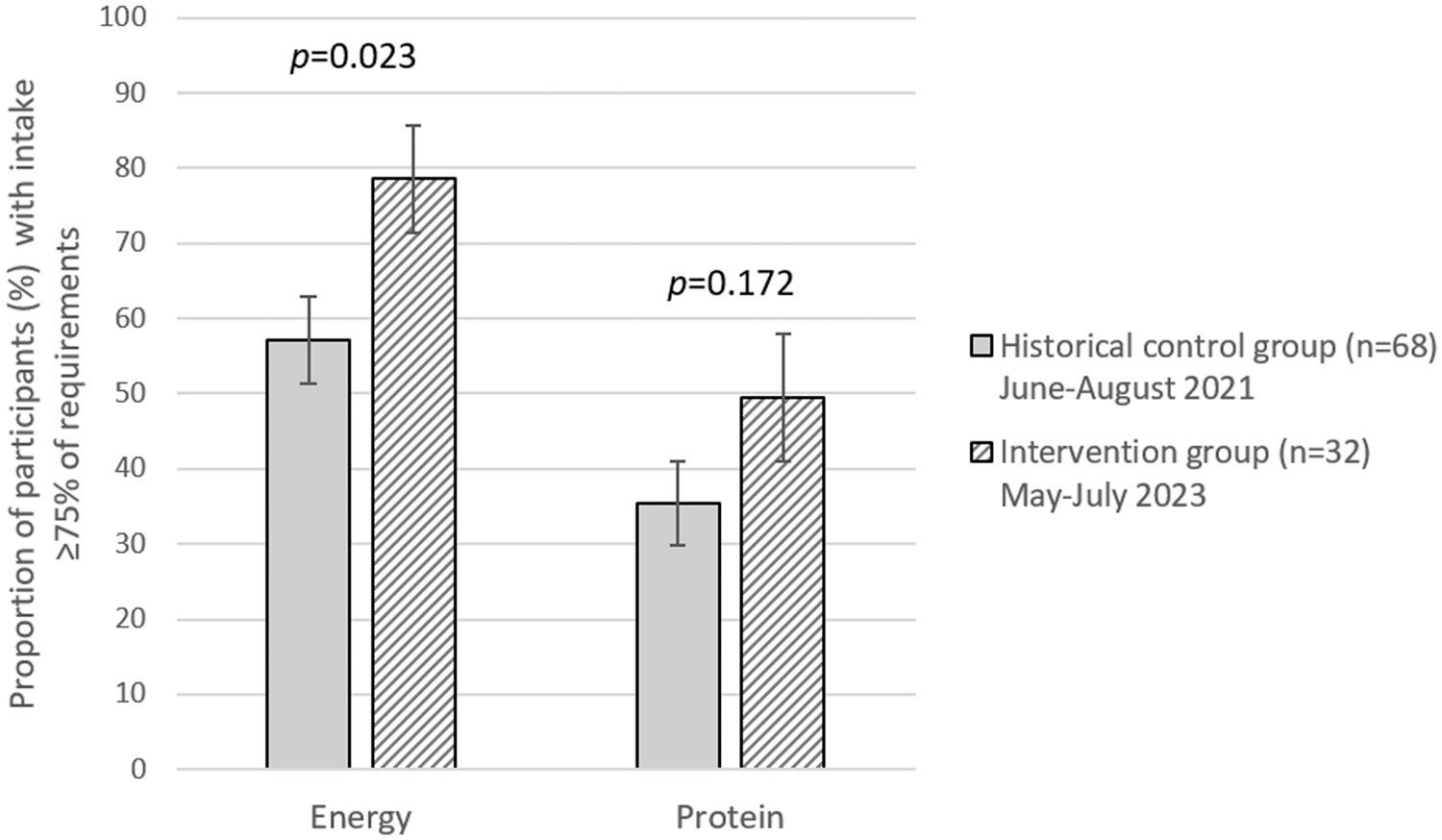
Energy and protein intake. Post‐estimated proportions (with standard errors) based on logistic regression models adjusted for gender, age, body mass index, and Nutritional Risk Screening 2002 Score. Group comparisons were made using a post‐estimation Wald test.

In the sensitivity analysis using standardised weight‐based energy requirements, the adjusted proportion of participants achieving at least 75% of estimated energy requirements remained higher in the intervention group than in the control group (76% vs. 59%, *p* = 0.052). Excluding participants with BMI < 18.5 kg/m^2^ yielded similar findings (75% vs. 50%, *p* = 0.016). A post hoc analysis applying the same exclusion to the primary energy analysis showed a similar pattern (84% vs. 56%, *p* = 0.003). Sensitivity analyses using standardised protein requirements of 1.2 and 1.3 g/kg/day showed a similar direction of effect but remained statistically non‐significant (1.2 g/kg/day: 48% vs. 39%, *p* = 0.406; 1.3 g/kg/day: 48% vs. 34%, *p* = 0.142).

### 3.3 Substudy 3: Nurses' and Clinical Dietitians' Perspectives

In substudy 3, nine female nursing staff members (eight nurses and one nursing assistant; all referred to as ‘nurses’) and five clinical dietitians (four female, one male) participated, with no invited participants declining. Interviews lasted 14–35 min.

All nurses had ≥ 2 years of professional experience (median 8.5 years), and clinical dietitians ≥ 1 year (median 2.5 years).

From the interviews, six themes were identified: two shared by both professional groups and two profession‐specific for each group (Figure 4).

**Figure 4:**
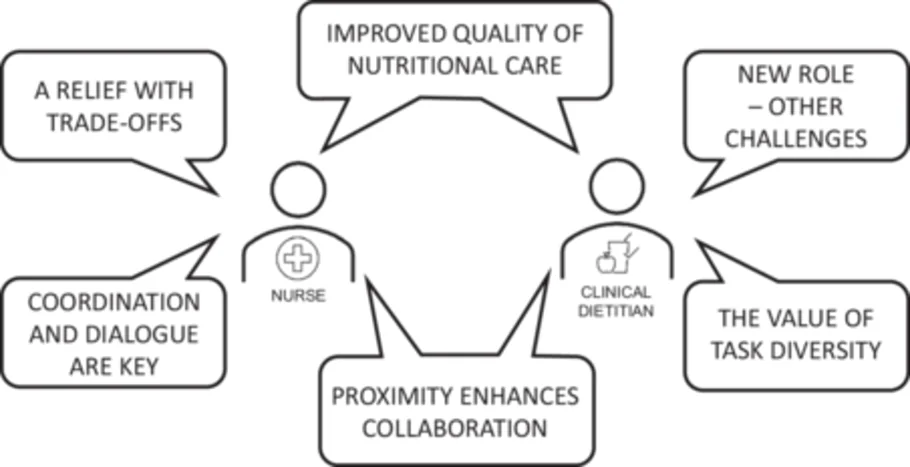
Identified themes in interviews with nurses and clinical dietitians. Themes were derived from interview transcripts using qualitative content analysis and reflect nurses' and clinical dietitians' experiences of a changed task distribution, including the reallocation of nutritional care tasks from nurses to clinical dietitians.

Both nurses and clinical dietitians experienced ‘*Improved quality of nutritional care’* during the intervention. Nurses described faster initiation of nutritional care, particularly enteral nutrition, and more consistent prioritisation.

I believe that the level – or rather, the nutritional care that the patients have received has improved. It has been better when dietitians have been involved than when we have to handle the task ourselves. But that's also because we have so many different care responsibilities to juggle, and we have to ask ourselves: “What comes first?” And yes, then nutrition sometimes ends up getting lost among all the other tasks.

(Nurse #3)

The presence of clinical dietitians at the ward provided new perspectives.

They see some things that we don't really think about, because we're not trained dietitians. We just have a bit of background from our nursing education, right, and it's not at all the same things we look at or look for.

(Nurse #2)

Clinical dietitians reported that ward presence improved monitoring and tailoring of nutritional care.

The advantage has been that you can follow the patient more closely … You are kind of seeing the same patient for several days in a row. Instead of just having 1 h during an on‐call visit, here you almost have 3 days. So you get to know the patient and their preferences.

(Clinical dietitian #3)

Under the theme ‘*Proximity enhances collaboration’*, both groups described how the clinical dietitian became a colleague rather than a guest, and how physical closeness fostered openness and collaboration.

Normally, you don't just call a dietitian for a quick discussion – there's also something relational about it when you don't really know who's on the other end … It really makes a difference knowing who it is … It's really nice to just be able to tap a colleague on the shoulder and say: “What should we do?”

(Nurse #8)

It leads to a much better dialogue, and you might feel more confident about sharing your opinions on various things than you would if you don't really know the people you're working with.

(Clinical dietitian #4)

Nurses described the assistance from clinical dietitians as a ‘*Relief with trade‐offs’*. On one hand, it eased their workload:

When we started getting help, we were in a very pressured situation in terms of resources, so it really – it was a gift to get assistance, because then we could be assured that the patients were getting the food they needed

(Nurse #1)

On the other hand, some nurses reported reduced focus on nutrition, declining skills, and a diminished sense of ownership:

I sometimes felt like I was lacking that feel for “what are they really eating?”

(Nurse #8)

These challenges could be addressed through better coordination and dialogue, which emerged as a separate theme from the nurses: ‘*Coordination and dialogue are key.’* Some noted that better communication could have prevented disruptions, for example, when meals were ordered without awareness of fluid restrictions or conflicted with nursing routines.

And sometimes it's also frustrating because if I have patients who can't eat on their own, and then I come in and the food has been ordered for 8 o'clock, but I'm not done administering medication and doing other tasks by then … I think there should have been better coordination, especially with regards to patients who need assistance.

(Nurse #7)

Clinical dietitians found the new tasks meaningful but also challenging, with the challenges relating less to their dietetic expertise than to the personal aspects of taking on a new role with patients. This was highlighted in the theme ‘*New role – different challenges’*.

It's an advantage that it's a dietitian who orders the food, even though it might not have been the task I personally found the most enjoyable… I think it was both in terms of the professional aspect and the personal one …being the first one in the morning to walk into a room with a possibly very sick patient was personally quite overwhelming for me.

(Clinical dietitian #4)

While recognising the value of the tasks, clinical dietitians emphasised ‘*The value of task diversity’* and did not want the role of ward‐based clinical dietitian to become their sole responsibility.

When you're in the outpatient clinic, you can have more in‐depth conversations with patients. In the medical pulmonary ward, it's a bit different; you're very close to the patient there. I really enjoy having a mix of these different roles as a dietitian.

(Clinical dietitian #2)

Both professional groups largely agreed on optimal allocation of nutrition‐related tasks (Figure 5). Nutritional risk screening and nutritional care plans were considered core responsibilities of the clinical dietitians, given their specialised knowledge.

We'll probably manage to carry out the initial screening; it's more the next steps that tend to fall through, because we get caught up in other tasks. So I think everything related to screening, developing a plan, initiating it – including having a dialogue with the patient and giving advice – that clearly belongs within the dietitian's area of expertise if we want the best possible quality.

(Nurse #1)

**Figure 5:**
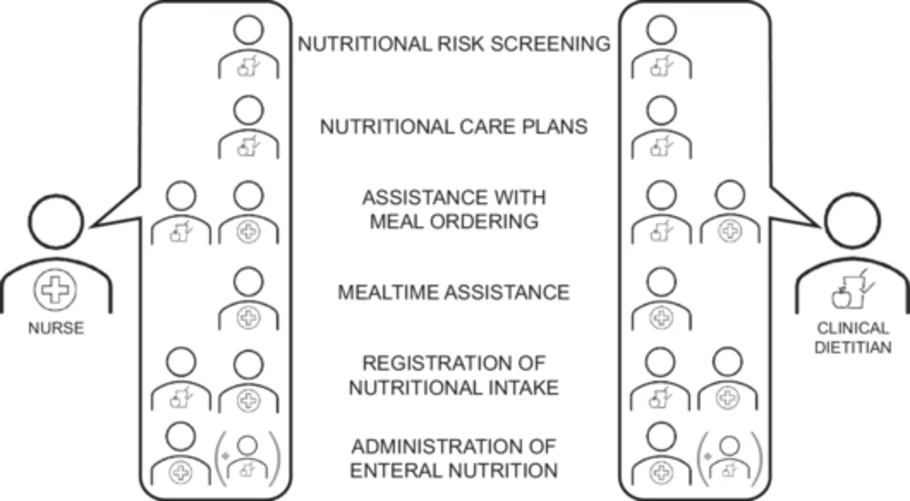
Nurses' and clinical dietitians' perspectives on optimal allocation of nutrition‐related tasks. Icons indicate which professional group(s) participants identified as most appropriate for each task, based on qualitative interviews. Shared icons denote shared responsibility.

Nutritional intake monitoring and assistance with meal ordering were considered shared responsibilities. Clinical dietitians were seen as particularly valuable for patients at nutritional risk because of their ability to provide tailored guidance and their familiarity with the foodservice system. However, as they were not present during evening hours and to maintain nurses' engagement in nutritional care, close collaboration was considered essential.

Both groups viewed mealtime assistance as the nurses' responsibility. Nurses emphasised the importance of maintaining a relational connection with patients during meals, while some clinical dietitians found this task too intimate.

I don't think it's something they necessarily need to take over. Because then I feel like we'd lose a part of the patient's care – especially when it comes to observations we need to make regarding whether the patient is able to eat their meals.

(Nurse #4)

Both groups agreed that, with appropriate training, clinical dietitians could assist with the administration of enteral nutrition.

## 4 Discussion

This multimethod study shows that reallocating nutritional care tasks from nurses to clinical dietitians was associated with improvements in nutritional care quality and was experienced as meaningful by both professional groups.

Importantly, the observed improvements should not be attributed to task reallocation alone. The intervention also involved daily ward presence of a clinical dietitian, allowing continuity of care, closer patient monitoring, and protected time for nutritional care. These organisational elements likely contributed to the observed changes and should be considered integral components of the model.

Nutritional risk screening rates were higher during clinical dietitian‐led care. Although this could potentially be attributed to the addition of a staff member, the reallocation was implemented in response to nursing shortages and, therefore, under conditions of overall staff scarcity. While similar improvements might theoretically be achieved by increasing nursing staff, our findings and previous research [9, 10, 11, 12] suggest that nurses face competing demands, with nutrition often deprioritised. Both nurses and clinical dietitians considered nutritional risk screening best performed by clinical dietitians. Supporting this, a recent study found limited agreement between nurses and clinical dietitians using NRS 2002, with misclassification by nurses associated with delayed dietitian involvement and longer hospital stays [24], while another Danish study reported improved screening and nutritional care following ward integration of clinical dietitians [13]. These findings support the involvement of clinical dietitians in nutritional risk screening to enable timely and appropriate nutritional care.

Despite improved screening rates, full guideline adherence was not achieved. This may partly reflect the inability to exclude admissions in short‐term beds, where screening is typically omitted. Although admissions lasting < 48 h were excluded, some expected short‐term stays were prolonged beyond 48 h and remained in the dataset.

In substudy 2, a greater proportion of intervention participants met ≥ 75% of energy requirements, whereas differences in protein intake were not statistically significant. This may partly reflect the limited sample size, as fewer patients were included in the intervention group despite a slightly longer recruitment period. The study may therefore have been underpowered to detect differences in protein intake. A retrospective sample size calculation based on the observed protein proportions suggested that approximately 263 participants in the historical control group and 124 in the intervention group would have been required to detect a difference of the observed magnitude with 80% power. The findings should therefore be considered hypothesis‐generating. In contrast, previous studies have demonstrated improvements in both energy and protein intake when á la carte foodservice systems are implemented as part of individualised care by clinical dietitians rather than as stand‐alone interventions [18, 25]. Findings from substudy 3 suggest potential mechanisms for this effect, as clinical dietitians were perceived as particularly valuable in supporting meal ordering for patients at nutritional risk through tailored guidance and effective use of the á la carte system, reflecting their ability to translate requirements into practical dietary strategies. Qualitative findings further highlighted complementary competencies between clinical dietitians and nurses, with dietitians contributing nutritional expertise and nurses contributing expertise in overall patient care.

The physical presence of clinical dietitians on the ward transformed their role from external consultants to team members, facilitating collaboration and improving nutritional care, consistent with previous qualitative studies [26, 27]. Furthermore, a Scandinavian survey found that nurses and physicians with greater access to clinical dietitians had a stronger focus on nutrition and found it easier to identify undernourished patients [28].

Concerns reported in earlier studies about increased nursing workload or overly complex treatment plans following clinical dietitian involvement [11, 26] were also reflected among a few nurses in this study. However, these challenges were described as opportunities for mutual learning, where dialogue enabled plans to be adapted to clinical realities, underscoring the importance of interprofessional collaboration in developing feasible, patient‐centred nutritional care strategies.

Although the task reallocation was largely viewed positively, some nurses experienced a sense of distancing from nutritional care. Future organisational models may therefore benefit from a more collaborative approach, in which clinical dietitians lead screening and care planning for patients at nutritional risk, while nurses remain actively involved in implementation and in supporting patients not at risk. Such models may preserve nursing engagement while optimising the use of professional expertise.

### 4.1 Strengths and Limitations

A key strength of this study is the triangulation of quantitative and qualitative methods. Although conducted in a single ward, the challenges addressed – such as competing nursing priorities, limited access to clinical dietitians, and suboptimal guideline adherence – are common across hospital settings, supporting broader relevance. Nevertheless, organisational structures and foodservice models differ between healthcare systems, and the findings should therefore be generalised with caution.

Some limitations should be acknowledged. In substudy 1, incomplete exclusion of admissions in short‐term beds may have affected screening estimates, although comparability between periods was maintained. Furthermore, screening practices during the post‐intervention period may have been influenced by the preceding intervention, and a carry‐over effect cannot be excluded. In substudy 2, the lack of inclusion flow data for the historical control group limits the interpretation of recruitment differences, and selection bias cannot be excluded despite adjustment for potential confounding. In addition, energy and protein requirements were estimated using different methods in the two study periods. However, these estimates represented the clinical targets used to guide nutritional care during each study period, meaning that the primary analyses reflect nutritional coverage relative to those treatment targets. Sensitivity analyses showed a similar pattern of findings, suggesting that the different estimation methods were unlikely to fully account for the observed between‐group differences. The larger between‐group difference after excluding participants with BMI < 18.5 kg/m^2^ was also observed using the original clinically estimated requirements, although the small number of participants with BMI < 18.5 kg/m^2^ (*n* = 24; 22% of the intervention group and 25% of the control group) limited further interpretation of this finding. Although the length of stay was reported descriptively, the study was not designed to evaluate the effect of the intervention on clinical outcomes such as length of stay, readmissions, or mortality, and no long‐term patient follow‐up was conducted. Consequently, it cannot determine whether the observed improvements in nutritional care translated into improved patient outcomes.

In substudy 3, the interviewer was a clinical dietitian employed at the hospital. While this may have facilitated rapport, it may also have influenced participants' responses through social desirability bias. Furthermore, participating nurses were identified with assistance from the head nurse, which may have introduced selection bias and influenced perceptions of voluntariness, although participation was voluntary. Although some interviews were relatively brief, the data were considered sufficient to address the research questions, as participants had direct experience with a focused intervention and additional interviews were not expected to provide substantially new insights. Conducting interviews during work hours may nevertheless have limited opportunities for more extensive reflection. Furthermore, the interviews were conducted shortly after completion of the intervention. While this timing enabled participants to reflect on their experiences while they were still recent, perceptions may have been influenced by the transition back to usual practice.

At the overall study level, the historical, non‐parallel design limits causal inference. Temporal changes in patient characteristics, staffing, clinical practice or other contextual factors between the comparison periods cannot be excluded and may have contributed to the observed differences. Although statistical adjustment and sensitivity analyses reduced some sources of bias, residual confounding remains possible. A parallel‐group comparison with individual‐level randomisation was inappropriate given the intervention's organisational nature. Future research should overcome these limitations by employing designs better suited for evaluating organisational interventions, such as stepped‐wedge cluster‐randomised controlled trials [29], which allow sequential implementation across wards while supporting stronger causal inference.

## 5 Conclusion

In conclusion, reallocating nutritional care tasks from nurses to clinical dietitians was associated with improvements in nutritional care and interdisciplinary collaboration and was experienced as meaningful by both professions. Ongoing coordination and dialogue remain essential, as effective nutritional care depends on close collaboration between clinical dietitians and nursing staff, regardless of how nutritional care tasks are allocated.

## Author Contributions

**Marianne Boll Kristensen:** conceptualisation, methodology, formal analysis, investigation, writing – original draft, visualisation, project administration, funding acquisition. **Ingeborg Krarup Rask:** methodology, formal analysis, writing – review and editing. **Lene Nissen Møller Schmidt:** resources, writing – review and editing. **Kirstine Guld Frederiksen:** conceptualisation, methodology, writing – review and editing. **Alexander Erichsen:** conceptualisation, methodology, investigation, writing – review and editing. **Lone Viggers:** conceptualisation, methodology, resources, writing – review and editing, funding acquisition.

## Funding

The authors have nothing to report.

## Conflicts of Interest

The authors declare no conflicts of interest.

## Supporting information


Supporting File 1

Supporting File 2

Supporting File 3

Supporting File 4

Supporting File 5

Supporting File 6

## Data Availability

The data that support the findings of this study are available on request from the corresponding author. The data are not publicly available due to privacy or ethical restrictions.
