# Supplementary material for: From Nurse‐Led to Clinical Dietitian‐Led Nutritional Care in Hospital Practice: Changes in Nutritional Risk Screening Rates, Patient Nutritional Intake and Professional Perspectives
Source: J Hum Nutr Diet. 2026 Oct 8;39(5):e70363. doi: 10.1111/jhn.70363 (PMC13650250; doi:10.1111/jhn.70363)
Supplement: Supplementary file 1 [file JHN-39-0-s001.docx]

**Supplementary file 6. Run chart of nutritional risk screening rates including admissions with a length of stay <48 hours (sensitivity analysis)**

The run chart shows the monthly proportion of admissions with nutritional risk screening completed within 24 hours of admission and overall, including admissions with a length of stay <48 hours. This sensitivity analysis was performed to assess the potential impact of excluding admissions with a shorter length of stay from the primary analysis. The sensitivity analysis included 3,634 admissions among 2,908 patients, compared with 1,678 admissions among 1,406 patients in the primary analysis.
