# Supplementary material for: From Nurse‐Led to Clinical Dietitian‐Led Nutritional Care in Hospital Practice: Changes in Nutritional Risk Screening Rates, Patient Nutritional Intake and Professional Perspectives
Source: J Hum Nutr Diet. 2026 Oct 8;39(5):e70363. doi: 10.1111/jhn.70363 (PMC13650250; doi:10.1111/jhn.70363)
Supplement: Supplementary file 2 [file JHN-39-0-s002.docx]

**Supplementary 5: Selected examples illustrating how subthemes and themes were derived through qualitative content analysis of transcripts of interviews with nurses and clinical dietitians**

| **Meaning units related to the research objective** | **Condensed meaning units abstracted into researchers own word** | **Subtheme** | **Theme** |
| --- | --- | --- | --- |
| *“They see some things that we don’t really think about, because we’re not trained dietitians. We just have a bit of background from our nursing education, right, and it’s not at all the same things we look at or look for.”* (Nurse #2) | Clinical dietitians identify different aspects compared to nurses. | An additional professional perspective | **IMPROVED QUALITY OF NUTRITIONAL CARE** |
| *“It has truly been a real gift to have yet another professional group here, because it just adds something—it's kind of like having a four-leaf clover. We, of course, already had nurses—so it becomes a five-leaf clover when there are doctors too—but then there were also occupational therapists, physiotherapists, and dietitians. It really adds something to have a broader, how can I say, a more complete picture of the whole patient and all aspects of their care.”*  (Nurse #8) | Inclusion of clinical dietitians enriches the multidisciplinary team. |  |  |
| *“Because you [clinical dietitians] come in with just that one focus, whereas we often have to keep an eye on: are they [patients] mobilized, do they have pressure ulcers, what about their nutrition, their pain, their breathing issues, and everything else, right?”*  (Nurse #1) | Clinical dietitians have nutrition as a dedicated focus, in contrast to nurses. | From competing demands to dedicated nutritional focus |  |
| *“I believe that the level – or rather, the nutritional care that the patients have received has improved. It has been better when dietitians have been involved than when we have to handle the task ourselves. But that’s also because we have so many different care responsibilities to juggle, and we have to ask ourselves: 'What comes first?' And yes, then nutrition sometimes ends up getting lost among all the other tasks.”* (Nurse #3) | Nutrition is often deprioritised among nurses’ competing priorities |  |  |

The table presents illustrative examples of the analytic process. Each subtheme and theme were derived from multiple meaning units across interviews, beyond those shown here.
