# Supplementary material for: From Nurse‐Led to Clinical Dietitian‐Led Nutritional Care in Hospital Practice: Changes in Nutritional Risk Screening Rates, Patient Nutritional Intake and Professional Perspectives
Source: J Hum Nutr Diet. 2026 Oct 8;39(5):e70363. doi: 10.1111/jhn.70363 (PMC13650250; doi:10.1111/jhn.70363)
Supplement: Supplementary file 3 [file JHN-39-0-s003.docx]

# Supplementary file 3: Standard tables used to estimate energy and protein requirements in the historical control group

**Estimation of daily energy requirements**

|  | **Goal: Weight maintenance** | | **Goal: Weight increase** | |
| --- | --- | --- | --- | --- |
| **Body weight (kg)** | *Bedridden*  *patients* | *Non-bedridden patients* | *Bedridden*  *patients* | *Non-bedridden patients* |
| **90** | 9.000 kJ/2.143 kcal | 10.000 kJ/2.381 kcal | 11.000 kJ/2.619 kcal | 13.000 kJ/3.095 kcal |
| **80** | 8.000 kJ/1.905 kcal | 9.000 kJ/2.143 kcal | 10.000 kJ/2.381 kcal | 13.000 kJ/3.095 kcal |
| **70** | 7.000 kJ/1.667 kcal | 8.000 kJ/1.905 kcal | 9.000 kJ/2.143 kcal | 11.000 kJ/2.619 kcal |
| **60** | 6.000 kJ/1.429 kcal | 7.000 kJ/1.667 kcal | 8.000 kJ/1.905 kcal | 10.000 kJ/2.381 kcal |
| **50** | 6.000 kJ/1.429 kcal | 7.000 kJ/1.667 kcal | 7.000 kJ/1.667 kcal | 9.000 kJ/2.143 kcal |
| **40** | 5.000 kJ/1.190 kcal | 6.000 kJ/1.429 kcal | 7.000 kJ/1.667 kcal | 8.000 kJ/1.905 kcal |
| *For patients with BMI>30 kg/m^2^* | 85 kJ/kg / 20 kcal/kg | 100 kJ/kg |  |  |

**Estimation of daily protein requirements**

|  | **Goal: Weight maintenance** | | **Goal: Weight increase** | |
| --- | --- | --- | --- | --- |
| **Body weight (kg)** | *Bedridden*  *patients* | *Non-bedridden patients* | *Bedridden*  *patients* | *Non-bedridden patients* |
| **90** | 95 g | 105 g | 115 g | 135 g |
| **80** | 85 g | 95 g | 105 g | 135 g |
| **70** | 75 g | 85 g | 98 g | 115 g |
| **60** | 65 g | 75 g | 85 g | 105 g |
| **50** | 65 g | 75 g | 75 g | 95 g |
| **40** | 55 g | 65 g | 75 g | 85 g |
| *For patients with BMI>30 kg/m^2^* | 0.9 g/kg | 1.1 g/kg |  |  |
