# Supplementary material for: From Nurse‐Led to Clinical Dietitian‐Led Nutritional Care in Hospital Practice: Changes in Nutritional Risk Screening Rates, Patient Nutritional Intake and Professional Perspectives
Source: J Hum Nutr Diet. 2026 Oct 8;39(5):e70363. doi: 10.1111/jhn.70363 (PMC13650250; doi:10.1111/jhn.70363)
Supplement: Supplementary file 4 [file JHN-39-0-s004.docx]

# **Supplementary file 4: Interview guide for semi-structured interviews with clinical dietitians and nurses regarding collaboration and task allocation in a pulmonary ward**

*The interview guide was translated from Danish into English for the purpose of this supplementary material.*

| **INTRODUCTION** | - Introduction of the interviewer - The interview is part of the evaluation of the collaboration between the Department of Nutrition and the pulmonary ward - AIM:   - To explore how clinical dietitians and nurses experienced the collaboration and task allocation   - To explore how clinical dietitians and nurses perceive individual nutrition-related tasks in relation to their professional role, competencies, and professional identity - The interview will be audio-recorded and transcribed - All information will be analysed and reported anonymously | |
| --- | --- | --- |
|  | | |
| **BACKGROUND INFORMATION** | - How long have you worked as a clinical dietitian/nurse? - How long have you worked in the pulmonary ward? | |
| **EXPERIENCES OF COLLABORATION** | For the past approximately 18 months, clinical dietitians have been an integrated part of the daily work in the pulmonary ward…   - Overall, how have you experienced this arrangement? - What advantages have you experienced? - What disadvantages have you experienced? - How have you experienced the collaboration with the clinical dietitians/nurses in the ward? - How have you made use of their professional expertise? - What have you learned from collaborating with clinical dietitians/nurses? | |
| **PERCEPTIONS OF TASK ALLOCATION IN RELATION TO PROFESSIONAL ROLE, COMPETENCIES AND PROFESSIONAL IDENTITY** | **CLINICAL DIETITIANS:**  As a clinical dietitian, you have been responsible for some tasks that have traditionally been performed by nursing staff, for example meal ordering and nutritional risk screening…   - How do you perceive the relevance of these tasks in relation to your professional role, competencies, and professional identity?   - In which tasks on the pulmonary ward did you make the greatest use of your dietetic expertise?     - How did you apply your professional expertise in these tasks?   - In which tasks did you make less use of your dietetic expertise?     - Why?   - Were there any tasks for which you felt less professionally prepared? If yes, which?   - Do you think you perform the tasks that were transferred to the clinical dietitians differently from how nursing staff perform them? - Compared with your other responsibilities as a clinical dietitian, how attractive did you find working in the pulmonary ward? - Which tasks did you particularly enjoy? - Which tasks did you enjoy less? - How frequently were you assigned to the pulmonary ward? - Would you have preferred to spend more or less time there? | **NURSES:** *The clinical dietitians have carried out some tasks that have traditionally been performed by nursing staff, for example meal ordering and nutritional risk screening…*   - How do you perceive the relevance of these tasks in relation to your professional role, competencies, and professional identity? - How has it affected you that you were less involved in these tasks while the clinical dietitians were working in the ward? - Which nutrition-related tasks do you consider most relevant to your profession and professional role?   - Why? - Which nutrition-related tasks do you consider less relevant to your profession and professional role?   - Why? - Are there any nutrition-related tasks for which you feel less professionally prepared? If yes, which? - Do you think you perform the tasks that were transferred to the clinical dietitians differently from how clinical dietitians perform them? |

| **FUTURE PERSPECTIVES** | *Nutritional care for hospitalised patients involves many different tasks, including nutritional risk screening, nutritional care planning, nutrition counselling, meal ordering, meal service, mealtime assistance, enteral feeding, and dietary intake monitoring.*  If you were establishing a completely new ward with unlimited resources (including unlimited numbers of nurses, clinical dietitians, and other healthcare professionals)…   - How would you organise responsibility for patients' nutritional care?   - Who should be responsible for which tasks (nutritional risk screening, nutritional care planning, nutrition counselling, meal ordering, meal service, mealtime assistance, enteral feeding, and dietary intake monitoring)?   - At what stage of the patient's admission?   - Why? (professional competencies, practical considerations, or other reasons?)   - Which nutrition-related tasks do you consider clinical dietitians and nurses to be most competent to perform?   - Would your answers differ for patients at nutritional risk compared with patients who are not at nutritional risk?   - How should interprofessional collaboration regarding patients' nutritional care be coordinated? |
| --- | --- |
|  | The current collaboration agreement ended in July 2023. If a similar collaboration were to be established in the future…   - Is there anything that should be done differently? |
| CLOSING | I have now asked about the topics I wanted to cover…   - Is there anything you would like to add? - Is there anything you think I should have asked about but did not? |
|  | |
| **DEBRIEFING** | - Thank you for sharing your perspectives - All information will be anonymised before analysis - During the analysis, we will look for themes that recur across interviews - If you have any questions, you are welcome to contact us |
