# Supplementary material for: From Nurse‐Led to Clinical Dietitian‐Led Nutritional Care in Hospital Practice: Changes in Nutritional Risk Screening Rates, Patient Nutritional Intake and Professional Perspectives
Source: J Hum Nutr Diet. 2026 Oct 8;39(5):e70363. doi: 10.1111/jhn.70363 (PMC13650250; doi:10.1111/jhn.70363)
Supplement: Supplementary file 5 [file JHN-39-0-s005.docx]

# Supplementary file 2: The electronic meal-ordering and nutritional intake registration system

Figure S2.1. Electronic meal-ordering interface

Patients use the photograph-based menu to select meals from those available within their prescribed diet. The menu is organised into food categories, and the figure shows selected dishes from the ‘*Desserts and snacks’* category. Text has been translated from Danish*.*

Figure S2.2. Nutritional intake registration interface

The interface allows registration of the amount consumed of each ordered food item using plate diagrams illustrating different proportions consumed. Foods and beverages consumed outside the electronic meal-ordering system can also be added. Text has been translated from Danish.

Figure S2.3. Nutritional intake overview

The figure shows the interface displaying recorded energy and protein intake. When individual energy and protein requirements have been entered into the system, nutritional intake is also visualised as a percentage of the estimated requirements. Text has been translated from Danish.
