# Supplementary material for: From Nurse‐Led to Clinical Dietitian‐Led Nutritional Care in Hospital Practice: Changes in Nutritional Risk Screening Rates, Patient Nutritional Intake and Professional Perspectives
Source: J Hum Nutr Diet. 2026 Oct 8;39(5):e70363. doi: 10.1111/jhn.70363 (PMC13650250; doi:10.1111/jhn.70363)
Supplement: Supplementary file 6 [file JHN-39-0-s006.docx]

**Supplementary file 1: Electronic nutritional risk screening form and screening status criteria used in data analysis**

The figure shows the electronic nutritional risk screening form used in the electronic patient record (translated from Danish).
In the assessment of nutritional status, predefined point values and corresponding descriptions within the three categories—weight loss, reduced nutritional intake, and low BMI—were presented as drop-down menus. Within each category, the healthcare professional was required to select the option yielding the highest score for the patient. Consequently, only the highest score from one of these three categories contributed to the nutritional status score.
By selecting *‘Calculate’*, the scores from the nutritional status assessment and the disease severity assessment were summed, with an additional point automatically added for patients aged ≥70 years.

For data analysis, classification of a patient as screened was based on the following criteria:

- First, it was assessed whether the *‘Primary nutritional risk screening’* checkbox had been selected. If the primary screening was marked but none of the associated statements were checked, the patient was classified as screened.
- If the primary screening was marked and one or more of the four statements were checked, completion of a secondary screening was required for the patient to be regarded as screened.
- A secondary screening was deemed completed if both the *‘Secondary nutritional risk screening’* checkbox and one response option under *‘Nutritional risk’* had been selected.
- Patients were also classified as screened if a secondary screening was conducted without a preceding primary screening.
